# Supplementary material for: Polyketide synthase gene clusters in the ericoid mycorrhizal fungus Oidiodendron maius: genome-wide prediction, expression profiling, and functional characterization of knockout mutants
Source: Front Plant Sci. 2026 Sep 16;17:1924430. doi: 10.3389/fpls.2026.1924430 (PMC13623903; doi:10.3389/fpls.2026.1924430)
Supplement: Supplementary file 1 [file SupplementaryFile1.zip › Supplementary Materials/Supplementary Folder 1.zip!/Oidma1_AssemblyScaffolds_Repeatmasked/knownclusterblast/cluster3/jgi.p_Oidma1_46824_mibig_hits.html]

| MIBiG Protein | Description | MIBiG Cluster | MiBiG Product | % ID | % Coverage | BLAST Score | E-value |
| --- | --- | --- | --- | --- | --- | --- | --- |
| BAQ25461.1 | putative\_dehydrogenase | BGC0001264 | Betaenone\_A\_/\_betaenone\_B\_/\_betaenone\_C\_biosynthetic\_gene\_cluster | 38 | 99.8394863563 | 373 | 4e-103 |
| ACA34720.1 | CtnD | BGC0000894 | Citrinin\_biosynthetic\_gene\_cluster | 29 | 100.963081862 | 211 | 2.6e-54 |
| ALI92648.1 | CitC\_oxidoreductase | BGC0001338 | Citrinin\_biosynthetic\_gene\_cluster | 29 | 100.963081862 | 211 | 2.6e-54 |
| AIG62134.1 | glucose-methanol-choline\_oxidoreductase | BGC0000120 | Patulin\_biosynthetic\_gene\_cluster | 28 | 102.247191011 | 187 | 4e-47 |
| EAU32818.1 | predicted\_protein | BGC0000160 | Terreic\_acid\_biosynthetic\_gene\_cluster | 28 | 99.6789727127 | 186 | 9e-47 |
| ctg1\_orf10 |  | BGC0000846 | Tabtoxin\_biosynthetic\_gene\_cluster | 36 | 53.290529695 | 168 | 2.5e-41 |
| CBF73676.1 | conserved\_hypothetical\_protein | BGC0001271\_c3 | Emericellin\_biosynthetic\_gene\_cluster | 28 | 101.926163724 | 163 | 6.2e-40 |
| EFG10343.1 | Glucose-methanol-choline\_oxidoreductase | BGC0000373 | Holomycin\_biosynthetic\_gene\_cluster | 30 | 58.7479935795 | 121 | 2.7e-27 |
| CAM56763.1 | hypothetical\_protein | BGC0000354 | Friulimicin\_biosynthetic\_gene\_cluster | 27 | 53.6115569823 | 109 | 1.4e-23 |
| ABY65992.1 | alcohol\_dehydrogenase | BGC0001008 | Maduropeptin\_biosynthetic\_gene\_cluster | 35 | 21.5088282504 | 86 | 1.3e-16 |
| AEF33092.1 | choline\_dehydrogenase | BGC0001039 | Pyridomycin\_biosynthetic\_gene\_cluster | 36 | 24.2375601926 | 74 | 5e-13 |
| ACH85567.1 | CetG | BGC0000283 | Cetoniacytone\_A\_biosynthetic\_gene\_cluster | 23 | 53.9325842697 | 69 | 2.1e-11 |
